# Pelvic osteotomies for acetabular dysplasia: Are there outcomes, survivorship and complication differences between different osteotomy techniques?

**DOI:** 10.1093/jhps/hnab009

**Published:** 2021-02-05

**Authors:** Edward C Beck, Anirudh K Gowd, Katlynn Paul, Jorge Chahla, Alejandro J Marquez-Lara, Jonathan Rasio, Torhu Irie, Joel Williams, Shane J Nho

**Affiliations:** 1 Department of Orthopedic Surgery, Wake Forest Baptist Health, Medical Center Blvd., Winston-Salem, NC 27157, USA; 2 Department of Orthopedic Surgery, Rush University Medical Center, 1611 W. Harrison St, Chicago, IL 60612, USA

## Abstract

The purpose of this study was to evaluate the safety and efficacy of Periacetabular osteotomy (PAO), rotational acetabular osteotomy (RAO), and eccentric rotational acetabular osteotomy (ERAO) for treating hip dysplasia by comparing complication rates, survivorship, and functional outcomes after treatment. A systematic review in the MEDLINE and CINAHL databases was performed, and studies reporting outcomes after pelvic osteotomy for hip dysplasia with a minimum of 1-year follow-up or reported postoperative complications was included. Patient demographics, radiographic measurements, patient reported outcomes including the modified Harris hip score (mHHS), complications using the modified Clavien-Dindo classification, and reoperations were extracted from each study. A meta-analysis of outcome scores, complications, change in acetabular coverage, and revision rates for the 3 pelvic osteotomies was performed. A total of 47 articles detailing outcomes of 6,107 patients undergoing pelvic osteotomies were included in the final analysis. When stratified by procedure, RAO had a statistically greater change in LCEA when compared to PAO (33.9° vs 18.0°; *P* <0.001). The average pooled mHHS improvement was 15.6 (95% CI: 8.3–22.8, *I*^2^= 99.4%). Although ERAO had higher mean score improvements when compared to RAO and PAO, the difference was not statistically significant (*P* >0.05). Lastly, patients undergoing PAO had a statistically greater complication rate than those undergoing ERAO and RAO (*P* <0.001 for both), while revision rate was not statistically different between the 3 techniques. In summary, there are many more publications on PAO surgery with a wide range of reported complications. Complications after ERAO and RAO surgery are lower than PAO surgery in the literature, but it is unclear whether this represents an actual difference or a reporting bias. Lastly, there are no significant differences between revisions, or postoperative reported outcomes between the 3 techniques.

## INTRODUCTION 

Hip dysplasia is an orthopaedic disorder that describes inadequate coverage of the femoral head in the hip socket. Due to the undercoverage, weight across the hip is distributed on a smaller surface area that results in excessive forces being placed on the hip joint [1]. The abnormal loading environment leads to early-onset degeneration of the hip joint, leading to cartilage wear and progression of early-onset osteoarthritis [2–4]. The periacetabular osteotomy (PAO) is the most commonly utilized method for addressing hip dysplasia worldwide [5], however, this technique is not commonly practiced in Asia where adult hip dysplasia is relatively common.

A common approach in Japan for addressing hip dysplasia in adolescents and young adults is the rotational acetabular osteotomy (RAO) [6], which was first described by Tagawa *et al*. [7] More recently, a modified procedure named the eccentric acetabular osteotomy (ERAO) was developed to prevent gluteus muscle weakness and limb-shortening associated with RAO [8]. While the differences in biomechanics and patient-reported outcomes of these two procedures have been evaluated in previous studies, they have not been extensively reported [9]. Furthermore, outcomes including patient-reported scores, hip-specific radiographic parameters and complications have not been compared between these two osteotomies and the PAO technique.

The purpose of this investigation was to evaluate the safety and efficacy of the three open surgical treatments used for hip dysplasia by assessing complications, radiographic parameters and short and mid-term outcomes after treatment. The authors hypothesized that (i) most complications would be neurologic in nature, (ii) survivorship following acetabular osteotomy will be high and (iii) patient-reported outcomes will be consistent across all three surgical techniques.

## MATERIALS AND METHODS

**Fig. 1.**
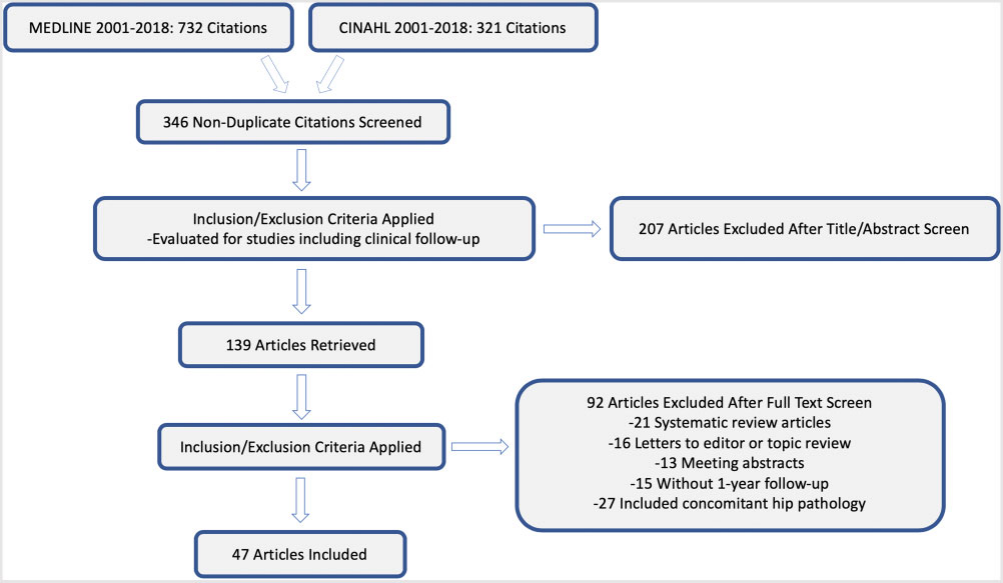
Flowchart demonstrating articles excluded as well as included in the final analysis. A total of 346 non-duplicate articles from the Medline and CINAHL databases were screened, with 52 included in the final analysis.

A systematic review of the MEDLINE and CINAHL databases was performed using PRISMA guidelines [10]. Systematic review registration was performed on 6 November 2018 using the PROSPERO International Prospective Register of systematic reviews (registration number CRD42018115942). Two independent reviewers (one board-eligible orthopaedic surgeon in sports medicine fellowship training and one orthopaedic research fellow) completed the search on 6 November 2018 and included studies between 1 January 2001 and 10 June 2018 using an explicit search algorithm: (“hip” AND [“PAO”, “RAO”, or ERAO”] AND “revision”) OR (“hip” AND [“PAO”, “RAO”, or ERAO”] AND “reoperation”) OR (“hip” AND [“PAO”, “RAO”, or ERAO”] AND “failure”) OR (“hip” AND [“PAO”, “RAO”, or ERAO”] AND “outcomes”) OR (“hip” AND [“PAO”, “RAO”, or ERAO”] AND “complications”). Studies that reported 1-year minimum outcome scores or complications of PAO, RAO or ERAO and included only patients with the diagnosis of hip dysplasia were included in the final analysis. Exclusion criteria included non-English language articles, participants with hip conditions other than hip dysplasia, studies that include other forms of surgical treatment for hip dysplasia and systematic reviews, meta-analyses or letters to the editor. Furthermore, electronically and printed journals were deemed acceptable, however, meeting abstracts and proceedings were omitted. All references within included studies were cross-referenced for potential inclusion if omitted from the initial search. The search algorithm used to generate the final studies for inclusion and analysis is provided in Fig. 1.

Each study was analysed for patient demographics, radiographic measurements, surgical procedure type, patient-reported outcomes, complications and survivorship following osteotomy for hip dysplasia. Data were collected for each procedure type independently and tabulated separately. Study demographics of interest included journal of publication, year of publication and level of evidence. Additionally, each study was also reviewed by two authors (E.C.B. and K.P.) for the Methodological Index for Non-randomized Studies (MINORS) criteria, which has been vetted as a reliable and valid assessment of reporting quality for both non-comparative and comparative outcomes studies [11]. Studies with a MINORS score of 13–16 for non-comparative studies or 21–24 for comparative studies were considered low risk of bias, while those with scores ≤12 for non-comparative studies or ≤20 for comparative studies were considered high risk of bias. Interobserver reliability was also calculated.

### Variables of interest

Patient demographics of interest included number of subjects, mean patient age and body mass index (BMI), gender frequency, mean follow-up time and specific population studied. Surgical technique data of interest included number of patients undergoing osteotomy. The primary outcome of interest was number of complications, and pre- and post-operative patient-reported outcomes including the Hip Harris Score (HHS), modified Hip Harris Score (mHHS), Hip Outcome Score (HOS), hip disability and osteoarthritis outcome score (HOOS) and Western Ontario and McMaster Universities Osteoarthritis Index (WOMAC), and number of revisions or conversions to total hip arthroplasty. Complications were recorded as both the specific type and the Clavien–Dindo classification modified for hip preservation surgery, which has been previously vetted and used for categorizing complications after hip surgery [12] (Table I).

**Table I.** List of Clavien–Dindo classification modified for hip preservation surgery^a^

Grade	Definition	Specific complications
I	A complication that requires no treatment and has no clinical relevance; there is no deviation from routine follow-up during the post-operative period; allowed therapeutic regimens include: antiemetics, antipyretics, analgesics, diuretics, electrolytes, antibiotics and physiotherapy	Asymptomatic Grade I or II heterotopic ossification; post-operative fever, nausea, constipation, minor UTI; wound problem not requiring a change in post-operative care
II	A deviation from the normal post-operative course (including unplanned clinic visits) that requires outpatient treatment: either pharmacologic or close monitoring as an outpatient	Superficial wound infection (additional clinic visits); transient neurapraxia from positioning or surgical retraction that resolves under close observation; nerve palsy requiring bracing and close observation (complete resolution); trochanteric delayed union
III	A complication that is treatable but requires surgical, endoscopic, or radiographic interventions or an unplanned hospital admission	Trochanteric nonunion; fracture; deep infection; surgical haematoma; clinically significant heterotopic ossification that requires surgical excision; deep vein thrombosis (admission and anticoagulation)
IV	A complication that is life threatening, requires ICU admission, or is not treatable with potential for permanent disability; a complication that requires organ resection (THA)	Osteonecrosis; permanent nerve injury; major vascular injury; pulmonary embolism; CNS complications; organ dysfunction
V	Death of a patient	

UTI, urinary tract infection; CNS, central nervous system; ICU, intensive care unit.

^a^ Adapted from Ref.^12^

### Statistics

Descriptive statistics were calculated for each study and variable or parameter analysed. Continuous variable data were reported as mean ± standard deviation. Categorical data were reported as frequencies with percentages. Meta-analysis was performed using the metaphor package of RStudio software version 1.0.143 (R Foundation for Statistical Computing). The DerSimonian–Laird estimator to determine effect sizes [13]. A fixed effects model was used for articles with low heterogeneity, while a random effects was used with high heterogeneity. Heterogeneity in pooled data was estimated using the *I*^2^ index. Forest plots were generated to display rates of revision hip surgery and conversion to hip arthroplasty. Predicted meta-regressions of adverse events between each surgical technique were compared using a Wald test [14]. Linear regression was used to determine significance in technique shifts. For all statistical analysis, *P* < 0.05 was deemed statistically significant. Publication bias was evaluated using a funnel chart. This plots the estimated treatment effect on the *x*-axis, while the size of each study is plotted on the *y*-axis. Larger studies are plotted at the top, while smaller on the bottom in order to characterize the respective effect sizes. Estimated effect sizes from each study were checked to be relatively evenly distributed and symmetrical around the treatment effect if little bias exists [15].

## RESULTS

Forty-seven articles detailing the outcomes of 5748 patients undergoing pelvic osteotomies were identified for the final analysis, including 25 PAO, 14 RAO and 8 ERAO studies. The studies used in the final analysis are outlined in Supplementary Appendix SI. There were no level-I studies, two level-II studies, 20 level-III studies and 25 level-IV studies. The average MINORS scores were 21.1 ± 1.6 and 15.4 ± 2.3 for comparative and non-comparative studies, respectively. There was a low risk of bias in 75% of comparative studies (6/8) and 92% of non-comparative studies (36/39). Interobserver reliability (κ) for MINORS score calculation was 0.78. The most frequent journals of publication were *Clinical Orthopaedics and Related Research* (14), *The Journal of Bone and Joint Surgery* (8) and the *Journal of International Orthopedics* (5). A funnel plot of effect sizes among all the studies in the analysis was constructed to evaluate publication bias/heterogeneity and noted to be relatively symmetrically distributed. However, some heterogeneity is still present due to differences in operative treatment (Supplementary Appendix SII).

Study and patient demographic data are presented in Table II. The combined mean age and BMI was 31.3 ± 7.4 and 23.1 ± 1.2, respectively, with a higher percentage of women (85%) undergoing pelvic osteotomies. The average age was slightly higher in the ERAO study groups (36.1 ± 4.1 years) when compared with PAO (27.1 ± 5.9 years) and RAO (35.9 ± 7.1 years). Comparison of BMI did not show any significant differences between the groups. The average follow-up was 94.8 ± 74.7 months.

**Table II.** Patient demographics

	PAO	RAO	ERAO	Total
Age	27.1 ± 5.9	35.9 ± 7.1	36.1 ± 4.1	31.3 ± 7.4
Gender				
Male	492 (16.1%)	139 (18.5%)	87 (8.8%)	718 (15%)
Female	2562 (83.9%)	613 (81.5%)	897 (91.2%)	4072 (85%)
BMI	23.7 ± 1.2	22.5 ± 0.85	22.0 ± 0.8	23.1 ± 1.2

### Radiographic findings

**Fig. 2.**
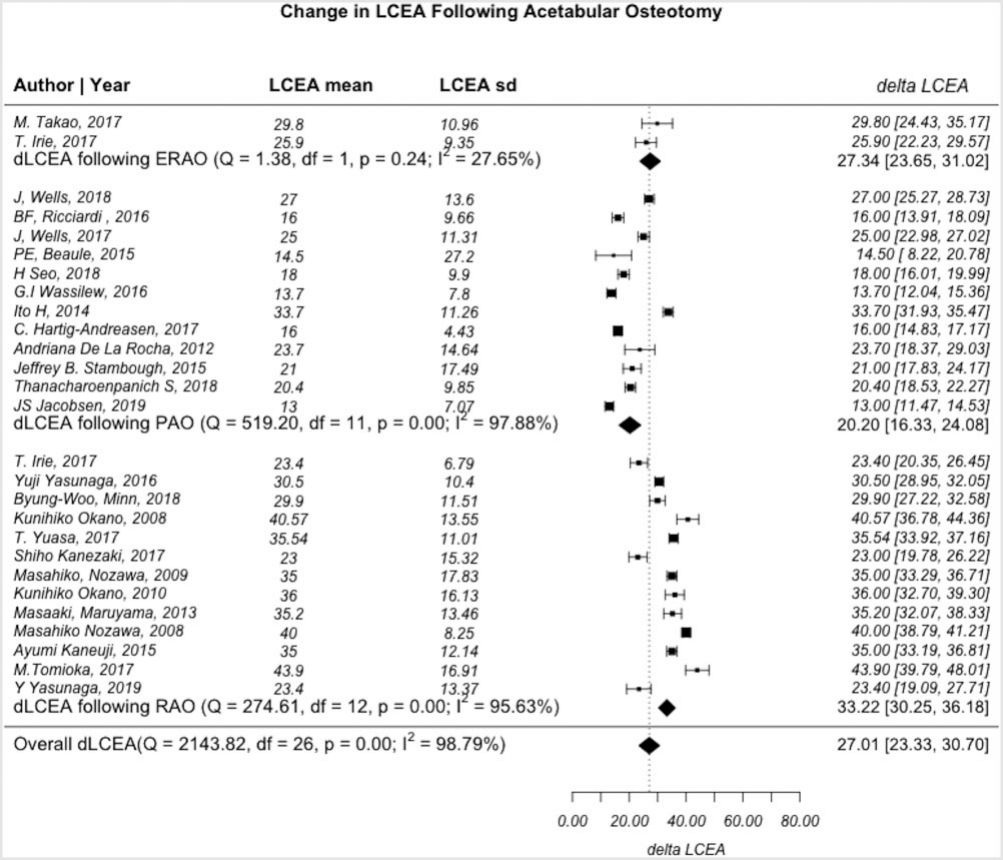
Forest plot demonstrating the heterogeneity of studies describing changes in lateral center edge angle before and after PAO, RAO and ERAO. *Q*-statistic: test of heterogeneity with *P* < 0.05 indicating significance. Df, degrees of freedom, defined as n−1; *I*^2^*,* measure of heterogeneity where values greater than 50% indicates heterogeneous findings.

A total of 27 articles reported lateral center edge angle (LCEA) (12 PAO, 2 ERAO and 13 RAO). The summary of pre- and post-operative LCEA measurements is provided in Table III. When stratified by procedure, the post-operative LCEA average for PAO and ERAO was 29.0 ± 6.8 and 32.6 ± 6.3, which are within the target LCEA of 25–35, while the RAO LCEA post-operative average of 36.9 ± 9.3 indicates some patients with acetabular overcoverage. The mean change was 20.2 [95% confidence interval (CI): 16.3–24.1, *I*^2^ = 97.9%] for PAO, 27.3 (95% CI: 23.7–31.0, *I*^2^ = 27.7%) for ERAO and 33.2 (95% CI: 30.3–36.2, *I*^2^ = 95.6%) for RAO. RAO had a statistically greater change in LCEA when compared with PAO (*P* < 0.001) (Fig. 2). However, there was no difference in LCEA change when comparing PAO versus ERAO and ERAO versus RAO (*P* < 0.05).

**Table III.** Radiographic parameters

	LCEA		Acetabular index	
	Pre- operative	Post- operative	Pre- operative	Post- operative
Combined	6.8 ± 9.1	33.1 ± 6.9	22.3 ± 7.5	4.7 ± 6.3
PAO	10.4 ± 9.9	29.0 ± 6.8	19.4 ± 7.6	5.4 ± 5.1
RAO	3.7 ± 8.6	36.9 ± 9.3	26.9 ± 7.3	5.8 ± 7.7
ERAO	4.8 ± 8.0	32.6 ± 6.3	22.3 ± 8.0	−2.8 ± 7.9

**Fig. 3.**
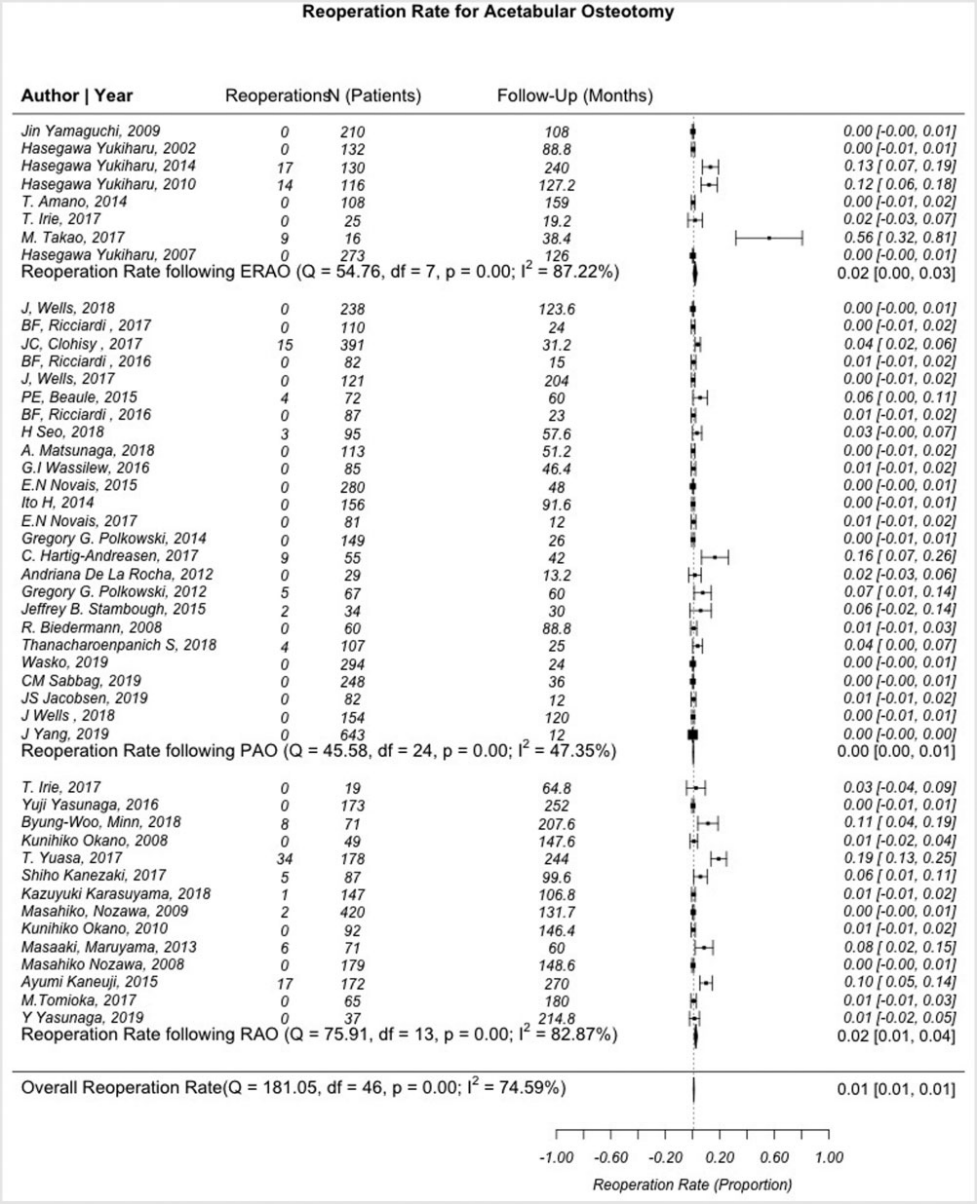
Forest plot demonstrating the heterogeneity of studies describing changes in the acetabular index or Tonnis angle before and after PAO, RAO and ERAO. *Q*-statistic, test of heterogeneity with *P* < 0.05 indicating significance; Df, degrees of freedom, defined as n−1; *I*^2^, measure of heterogeneity where values greater than 50% indicates heterogeneous findings.

Of the studies reporting radiographic measurements, 19 reported change in acetabular index (10 PAO, 7 RAO and 2 ERAO). When stratified by procedure, the post-operative acetabular index average for PAO and RAO was 5.4 ± 5.1 and 5.8 ± 7.7, which are within the target acetabular index of 0–1, while the ERAO post-operative acetabular index −2.8 ± 7.9 indicates some patients with acetabular overcoverage. When stratified by procedure, mean change was −12.9 (95% CI: −15.9 to −9.9, *I*^2^ = 97.9%) for PAO, −24.6 (95% CI: −29.9 to −19.4, *I*^2^ = 54.3%) for ERAO and −20.9 (95% CI: −25.2 to −16.6, *I*^2^ = 96.0%) for RAO (Fig. 3). Comparison of the changes in acetabular index angle demonstrated that RAO and ERAO had a statistically greater change as compared to PAO (*P* < 0.001).

### Reported outcomes

**Fig. 4.**
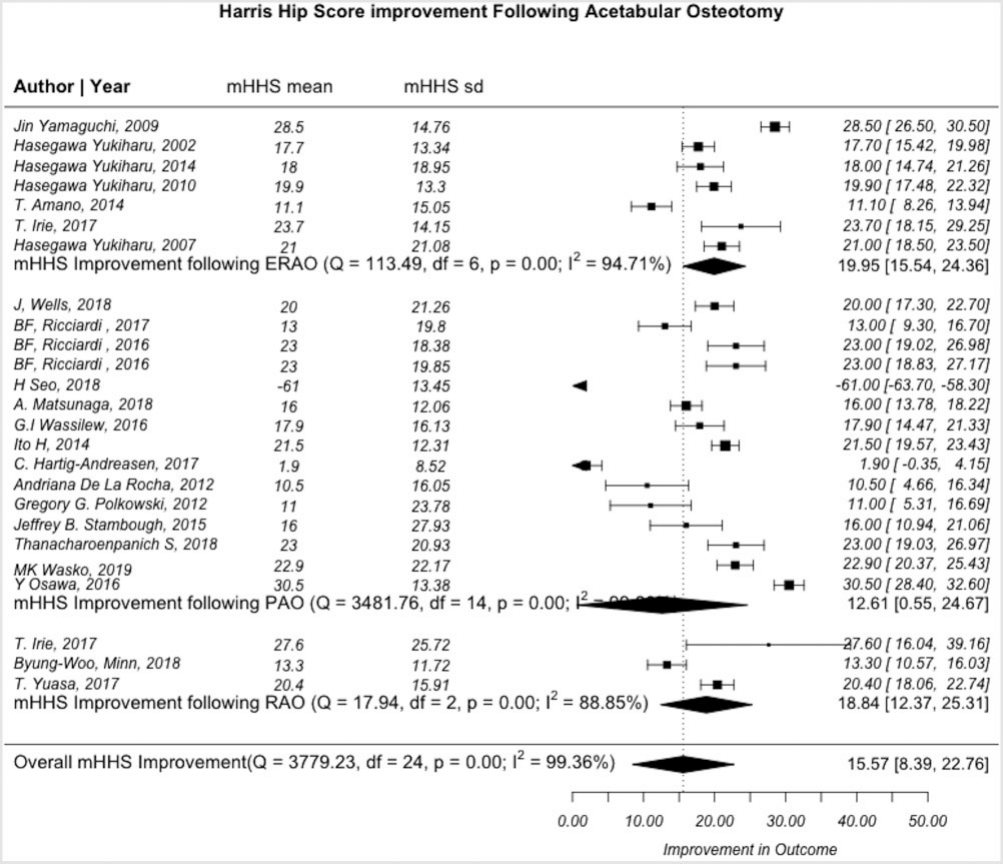
Forest plot demonstrating the heterogeneity of studies describing changes in the Hip Harris score or modified Hip Harris score before and after PAO, RAO and ERAO. *Q*-statistic, test of heterogeneity with *P* < 0.05 indicating significance; Df, degrees of freedom, defined as n−1; *I*^2^, measure of heterogeneity where values greater than 50% indicates heterogeneous findings.

The most consistent reported outcome used in pelvic osteotomy studies was the HHS and mHHS, which were subsequently were used for the final analysis. Twenty-five articles reported HHS or mHHS (15 PAO, 7 ERAO and 3 RAO), with a pre- and post-operative mean of 67.0 ± 11.6 and 83.04 ± 12.5, respectively (Table IV). The average pooled HHS and mHHS improvement was 15.6 (95% CI: 8.3–22.8, *I*^2^ = 99.4%). When stratified by procedure, the mean HHS and mHHS improvement was 20.0 (95% CI: 15.5–24.4, *I*^2^ = 94.7%) for ERAO, 12.6 (95% CI: 0.4–24.8, *I*^2^ = 99.6%) for PAO and 18.8 (95% CI: 12.4–25.3, *I*^2^ = 88.9%) for RAO (Fig. 4). There was no statistically significant difference between HHS and mHHS improvement between surgical techniques (*P* > 0.05).

**Table IV.** Comparison of baseline and post-operative mHHS/HHS scores

	Baseline Score	Post-operative Score	P-value
PAO	65.9 ± 11.8	79.2 ± 11.7	<0.001
RAO	67.5 ± 7.9	87.3 ± 10.3	<0.001
ERAO	69.5 ± 9.9	89.5 ± 12.1	<0.001

### Complication rates

Thirty-three articles, including a total of 4883 patients, were available for complication rate analysis (21 PAO, 9 RAO and 3 ERAO). The list of complications by type and Clavien–Dindo Grade is summarized in Table V. Briefly, there were a total of 14.1%, 3.1% and 12.6% complications reported in the PAO, RAO and ERAO patient group, respectively. When comparing by complication type, neuropathy and other nerve damage was the most common complication in PAO procedures (2.9%), while the formation of heterotopic ossification was the most common complication for both RAO and ERAO procedures (1.2% and 1.9%).

**Table V.** List of post-operative complications

	PAO	RAO	ERAO	Total
Total complications	464 (14.1%)	37 (3.1%)	53 (12.6%)	554 (11.3%)
Modified Clavien–Dindo system				
Grade I	181	18	12	211
Grade II	123	0	22	145
Grade III	120	19	18	157
Grade IV	39	0	1	40
Grade V	0	0	0	0

**Fig. 5.**
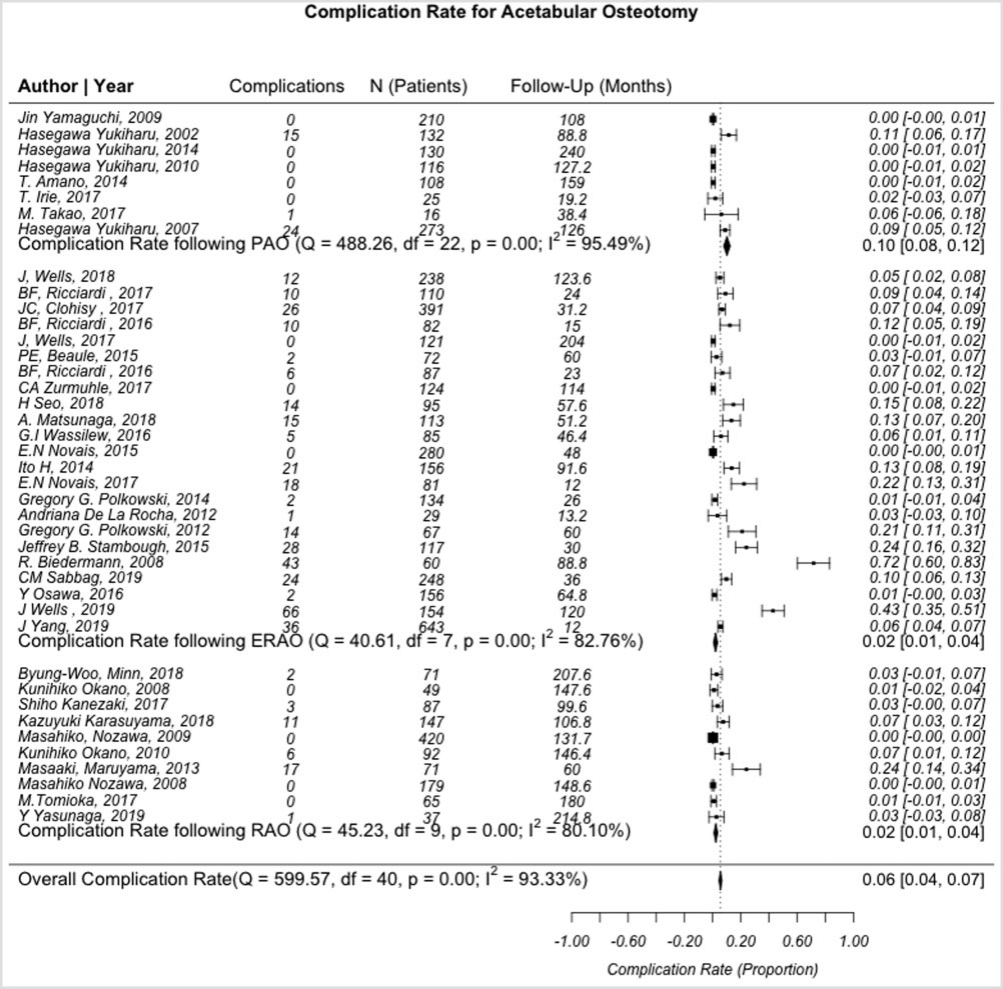
Forest plot demonstrating the heterogeneity of studies describing complication rates after PAO, RAO and ERAO. *Q*-statistic, test of heterogeneity with *P* < 0.05 indicating significance; Df, degrees of freedom, defined as n−1; *I*^2^, measure of heterogeneity where values greater than 50% indicates heterogeneous findings.

Pooled complication rates for PAO, RAO and ERAO were 0.10 (95% CI: 0.08–0.12, *I*^2^ = 95.5%), 0.02 (95% CI: 0.01–0.04, *I*^2^ = 80.1%) and 0.02 (95% CI: 0.01–0.04, *I*^2^ = 95.5%), respectively (Fig. 5). Analysis of the reported complications demonstrated that patients undergoing PAO and ERAO had a statistically greater complication rate than those undergoing RAO (*P* < 0.001 and *P* = 0.004, respectively). While PAO had a higher frequency of reported complications versus ERAO, the difference was not statistically significant (*P* = 0.271).

### Reoperation rates

**Fig. 6.**
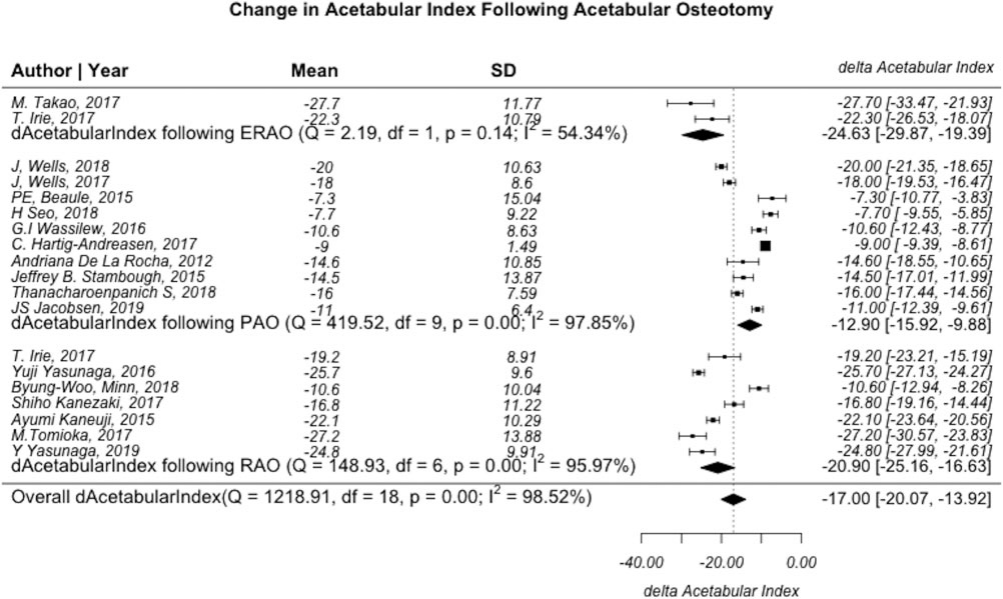
Forest plot demonstrating the heterogeneity of studies describing rates of reoperation after PAO, RAO and ERAO. *Q*-statistic: test of heterogeneity with *P* < 0.05 indicating significance. Df, degrees of freedom, defined as n−1; *I*^2^, measure of heterogeneity where values greater than 50% indicates heterogeneous findings.

Forty-seven articles, including a total of 5871 patients, were available for reoperation rate analysis (25 PAO, 14 RAO and 8 ERAO). Reoperations were defined as either revision osteotomy or conversion to THA. The combined reoperation rate was 2.5%, with reoperation rates for PAO, RAO and ERAO being 1.1% (*I*^2^ = 47.4%), 5.1% (*I*^2^ = 82.9%) and 4.1% (*I*^2^ = 87.2%), respectively (Fig. 6). Patients undergoing PAO had a statistically lower rate of reoperation when compared with both RAO and ERAO (*P* < 0.001 for both), while the difference between RAO and ERAO was not statistically significant (*P* values = 0.288).

## DISCUSSION

The main findings of the current meta-analysis were that there are no statistically significant differences in reported outcomes in patients undergoing PAO, RAO or ERAO for hip dysplasia. Furthermore, current literature does not demonstrate any significant differences between revisions between the three acetabular osteotomy types. However, patients undergoing RAO have a statistically significant lower rate of reported complications when compared with patients undergoing PAO and ERAO, with neuropathy being the most common type among PAO procedures. While these procedures are distinct in their approach and technique, and there are measurable differences in outcomes, all three have comparable high success rates. Pelvic osteotomies utilized for the management of hip dysplasia remain a challenging procedure with a steep learning curve for orthopaedic surgeons. While numerous techniques have been described, the goal of an ideal osteotomy is to be as soft tissue sparing as possible, reproducible, allow sufficient correction to maximize femoral head coverage and weight bearing hyaline cartilage area while avoiding impingement, have reliable blood supply for healing and result in a stable osteotomy.

Pelvic osteotomies are invasive procedures and can be associated with major and minor complications [16]. In this review, RAO was associated with a lower reported rate of overall complications (3.1%) as compared to PAO (14.1%) and ERAO (12.6%). There is a potential reporting bias as only three ERAO papers reported complications, a bit more than half of the RAO papers, but almost all PAO papers. Neuropathy (2.9%) was the most common complication for PAO followed by HO formation and delayed union. While Cates *et al*. [17], reported a rate of 90% for lateral femoral cutaneous nerve (LFCN) dysesthesia following PAO, all other publications have reported much lower rates [18]. Given that some variation of an anterior approach to the hip is most often utilized to perform a PAO, it is not surprising that nerve injury is frequently cited as the most common complication. An anterior approach to the hip places the LFCN at risk of injury as it courses in the deep layer of the subcutaneous fat tissue along the lateral aspect of Sartorius muscle. However, the majority of LFCN dysesthesia resolve over time and many surgeons may not report them as complications. Nerve related complications for RAO and ERAO, were much lower (0.33% and 0.29%, respectively), which is likely related to the lateral approach utilized in these techniques, helping to avoid injury to the LFCN. RAO utilizes a lateral based skin incision and then combines an anterior and posterior inter-nervous plane approaches to the hip [19]. ERAO typically utilizes a trans-trochanteric lateral approach to the hip, while limiting the anterior end of the incision to decrease the risk of the LFCN [20]. Despite these technical differences, standardizing the reporting of nerve injury following acetabular osteotomies may help minimize the variability in reporting of this common complication and its impact on patient outcomes.

Heterotopic bone formation was the most common complication reported for both RAO and ERAO (1.2% and 1.9%). A similar rate of 2.7% was also reported for PAO. Wells *et al*. [18], recently reported a rate of HO as high as 34.4% following PAO, the majority of which were asymptomatic (Brooker Class I–II). HO is a well-recognized phenomenon in approaches to the acetabulum, particularly following trauma [21]. Although the pathophysiology of HO formation is not completely understood, it is generally accepted that a permissive environment of increased inflammation is necessary to stimulate osteoprogentior cells from tissues, such as muscle and periosteum or stromal cells from the bone marrow [22]. With any of the open techniques, muscles may undergo extensive and prolonged retraction during surgery potentially causing local tissue ischaemia/necrosis and worsening inflammation. HO has been reported to occur most commonly along with gluteus minimus and iliocapsularis muscle after PAO surgery and within the gluteus medius and minimus after RAO/EROA surgery [23]. As such care must be taken to avoid forceful retraction or injury to these muscles and modifications of the surgical approaches and instrumentation have evolved to minimize soft tissue damage.

The contemporary rectus sparing version of the Smith–Peterson approach is utilized most often for PAO, which is more muscle sparing than the trans-trochanteric technique used for RAO and ERAO. The initial procedure described by Mast and has evolved somewhat since the original description. The elegant exposure is inter-muscular and inter-nervous, which minimizes disruption of the TFL, gluteus minimus and iliocapsularis. The direct head of the rectus femoris historically was released from the anterior inferior iliac spine but is rarely, if ever, needed with contemporary technique [5]. Non-steroidal anti-inflammatory drugs may help reduce the risk of HO, however, long-term utilization of NSAIDs (>6 weeks) has been associated with delayed union of acute acetabular fractures and should be used with care [24].

Reporting of delayed union was also lower for RAO and ERAO 0.57% and 0.59%, respectively, compared to PAO (1.81%). RAO and ERAO are osteotomies allow for increased surface area of bone contact between the acetabular segment and the remaining intact pelvis, which in theory may be more advantageous for bone healing. However, as part of the osteotomy is intraarticular and the articular segment is small, there is a very worrisome potential for vascular compromise to the articular segment which can result in avascular necrosis, chondrolysis and arthrosis [25]. In the case of ERAO, the space between the osteotomized acetabular segment and the pelvis is minimal, often not requiring a bone graft, which is a major advantage over the RAO [8].

It is worth noting that there is variability in methods of reporting complications between studies, which may account for the differences observed in rates of delayed union, as well as other complications among the three techniques. Recently, Sink *et al*. [12] proposed using a modified version of the Clavien–Dindo classification for reporting post-operative complications related to hip surgery. Very few of the studies in the current review reported complications using this classification system, and of those who did, most reported only major complications (defined as Grade III/IV). It is possible that reporting bias due to underreporting of minor complications by patients or clinicians due complete or partial symptom resolution may occur [26]. This highlights the need for more robust prospective studies evaluating the rates of post-operative complications and using standardized classification systems for reporting them.

Radiographic parameters demonstrated possible overcorrection in some cases for both ERAO and RAO. Based on the modern understanding of acetabular coverage of the femoral head [27], the LCEA average in the RAO and Acetabular Index in the ERAO groups indicates that many patients had post-operative acetabular overcoverage. Previous studies have shown that impingement caused by acetabular overcoverage leads to premature joint degeneration [28]. However, it is worth noting that there is some variability in post-operative target angle, with some authors reporting on RAO outcomes defining non-overcorrection as an LCEA of ≤40° [29]. In other studies, authors have determined post-operative target angles to be patient-specific; balancing femoral head coverage and bone contact area, with an LCEA range of (20°–46°) [28]. The differences in post-operative target angle could account for the differences observed in post-operative angles between the three. Additionally, some other factors including variability in volume socket or sourcil size could have limited the ability to achieve target angles.

Irie *et al*. [9] compared the Harris Hip score averages of patients undergoing RAO or ERAO for hip dysplasia and demonstrated that patients in the ERAO group had statistically higher score averages when compared with the RAO group [93.6 (95% CI 70–100) versus 89.7 (95% CI 74–100); *P* = 0.09]. However, the authors noted that the groups were very small (*N* = 17 and *N* = 22) and the follow-up was shorter in the ERAO group which likely influenced the observed differences in score averages. In contrast, the present review demonstrated no statistical difference in mHHS or HHS scores between RAO, ERAO and PAO over an average follow-up of 85 months indicating that radiographic parameters alone may not play a role in patient-reported outcomes following osteotomy procedures for hip dysplasia. This highlights the need for further studies evaluating the effect of overcoverage on outcomes among patients undergoing acetabular osteotomy.

A limited number of studies have reported on the long-term survivorship following corrective acetabular osteotomies. Overall, the present review demonstrated a lower reoperation rate among patient undergoing PAO when compared with RAO and ERAO. When specifically looking at the original Bernese PAO cohort, conversion to THA, arthritis progression and/or Merled’Aubigne’–Postel score <15 were 82%, 60% and 43% at 11-, 20- and 30-year follow-up, respectively [30–32]. In a more recent systematic review, Sohatee *et al*. [33] observed a THA conversion rate of 8.3% among 4862 patients, with a mean conversion time of 5.8 years after undergoing PAO. At an average of 11–13 years following RAO, the survival rate with THA as an endpoint has been reported to range between 95% and 100% [34]. Following ERAO Hasegawa *et al*. [20], noted a survival rate of 87% at 20 years. Of the 17 patients (13%) that underwent THA in this series, 11 had advanced stage osteoarthritis pre-operatively and 6 had undergone simultaneous intertrochanteric valgus osteotomy. Osteoarthritis is a well-recognized risk factor for failed acetabular osteotomy and progression of disease. However, Nozawa *et al*. [35] demonstrated that in younger patients (age ≤ 60 years) with hip dysplasia and advanced osteoarthritis, RAO was associated with improved pain and function and a rate of conversion to THA of 12.3% after an average follow-up of 12.2 years. Interestingly, 45.6% of the hip demonstrated progression of OA based on radiographic parameters.

The current literature does not appear to demonstrate improved longevity of one osteotomy over the other, however, variability in patient populations, cultural perceptions of pain, associated hip pathology and cultural views of and access to arthroplasty limits the ability to compare long-term outcomes and conversion to THA. While better quality studies are warranted, the data suggest that all three acetabular osteotomy techniques are a safe and effective surgical option for patients with hip dysplasia and early osteoarthritis and provide high rates of survivorship at long-term follow-up.

### Limitations

There are limitations to this study that should be addressed. First, the majority of studies analysed were retrospective case series, which introduces an inherent selection bias. Second, this study gathered data from a highly heterogeneous group of subjects (demographics, cultures, radiographic parameters, indications for surgery and follow-up time) who have undergone pelvic osteotomy, which may include variables that impact outcomes. Additionally, while there were only three techniques compared in this meta-analysis, there may be surgeon-specific variability due to technique, experience, or number of cases performed per year that may influence outcomes. Third, due to the way that the data are analysed, it is difficulty to perform subgroup analyses, which may limit the generalizability of the results. Fourth, although there are a number of newer hip-specific questionnaires available for evaluating hip function, many were not yet created when many of the studies were performed. As such, many studies reported the modified Hip Harris score, which is commonly used to evaluate older arthritic patients. It is possible that other questionnaires may be better suited for evaluating hip function in patients after undergoing acetabular osteotomy. Lastly, although there was a low risk of bias in both comparative and non-comparative studies, there was variability in the type of published reports and a large number of low level of evidence studies included in the meta-analysis.

## CONCLUSION

There are no statistically significant differences in reported outcomes in patients undergoing PAO, RAO or ERAO for hip dysplasia. Furthermore, current literature does not demonstrate any significant differences between revisions between the three acetabular osteotomy types. The only statistically significant difference was a higher rate of reported LCFN dysfunction when compared with patients undergoing RAO or ERAO that is typically transient, does not affect hip function or longevity of the procedure. The limited number of reported complications in the ERAO and RAO publications suggests that there is likely a difference in reporting. Future, long-term studies are warranted to improve the quality of the literature regarding the management of complex hip pathology.

## SUPPLEMENTARY DATA

Supplementary data are available at *Journal of Hip Preservation Surgery* online.

## Supplementary Material

hnab009_Supplementary_Data
