# Supplementary material for: Pelvic osteotomies for acetabular dysplasia: Are there outcomes, survivorship and complication differences between different osteotomy techniques?
Source: J Hip Preserv Surg. 2021 Feb 5;7(4):764–76. doi: 10.1093/jhps/hnab009 (PMC8349594; doi:10.1093/jhps/hnab009)
Supplement: hnab009_Supplementary_Data [file hnab009_supplementary_data.zip › hnab009-suppl_data/Appendix I.docx]

| First Author | Journal | Year | Surgery | Number of Patients | Number of Hips | Mean Follow Up Time | Age | BMI | Reoperations | Baseline mHHS/HHS | Postop mHHS | Complications |
| --- | --- | --- | --- | --- | --- | --- | --- | --- | --- | --- | --- | --- |
| Yukiharu H | CORR | 2007 | ERAO | 248 | 273 | 126 | 29.7 | 21.8 | 9 | 71+9.5 | 91+16.2 | 26 |
| Yamaguchi J | CORR | 2009 | ERAO | 210 | - | 108 | 40.8 | - | 0 | Old: 60.2+8.3 | 88.7+13 | - |
|  |  |  |  |  |  |  |  |  |  | Young:63.1+8.8 | 92+11.7 | - |
| Amano T | Hip Int | 2014 | ERAO | - | 530 | 159 | 35.9 | 21.9 | 0 | Male:74.6+7.3 | 94.1+7.3 | - |
|  |  |  |  |  |  |  |  |  |  | Female: 74.0+5.6 | 93.5+11.3 |  |
| Irie T | Hip Int | 2017 | ERAO | 17 | 19 | 19.2 | 32.6 | - | 0 | 69.9+9.8 | 93.6+3.8 | - |
| Takao M | Int J of Computer Assisted Rad and Surg | 2017 | ERAO | 15 | 16 | 38.4 | 33.7 | 22.3 | 0 | - | - | 1 |
| Yukiharu H | Journal of Orthopaedic Science | 2010 | ERAO | 113 | 116 | 127.2 | 42.2 | 22.2 | 14 | 65 | 88 | - |
| Yukiharu H | JBJS | 2002 | ERAO | 126 | 132 | 88.8 | 36.5 | - | 0 | 71.3+8.1 | 89.0+10.6 | 25 |
| Yukiharu H | JBJS | 2014 | ERAO | 124 | 130 | 240 | 37.4 | 21.8 | 17 | 70+9.8 | 88.0+16.3 |  |
| Jacobsen JS | Acta Orthopaedica Scandinavica | 2019 | PAO | 82 | 82 | 12 | 30.5 | 23.5 | 0 | - | - | - |
| Ito H | BMC Musculoskeletal Disorders | 2014 | PAO | 138 | 156 | 91.6 | 30 | - | 0 | 68.5+8.4 | 90.0+9.0 | 21 |
| Wassilew GI | Cell Tissue and Banking | 2016 | PAO | 73 | 85 | 46.4 | 28.3 | - | 0 | 70.2+11.0 | 88.1+11.8 | 5 |
| De La Rocha A | CORR | 2012 | PAO | 26 | 29 | 13.2 | 16.3 | - | 0 | PPSx:65.9+12.1 | 78.5+10.4 | 1 |
|  |  |  |  |  |  |  |  |  |  | NPSx:65.6+9.5 | 75.6+12.3 |  |
| Novais EN | CORR | 2017 | PAO | - | 81 | 12 | 18 | 23 | 0 | - | - | 5 |
| Polkowski GG | CORR | 2012 | PAO | 63 | 67 | 60 | 19.2 | 23 | 2 | 70.0+18.3 | 81.0+15.3 | 12 |
| Polkowski GG | CORR | 2014 | PAO | 134 | 149 | 26 | 30 | 25 | 0 | - | - | 2 |
| Wells J | CORR | 2019 | PAO | 129 | 154 | 120 | 26 | 24 | 0 | - | 86.1+15.7 | 66 |
| Wells J | CORR | 2017 | PAO | 99 | 121 | 204 | 28.3 | 26.3 | 0 | - | 65.0+13.0 | - |
|  |  |  |  |  |  |  |  |  |  |  |  |  |
| Stambough JB | CORR | 2015 | PAO | 39 | 39 | 30 | 19.7 | 23.6 | 4 | Study:62.0+18.9 | 78.0+18.4 | 9 |
|  |  |  |  |  |  |  |  |  |  | Control:59.0+23.6 | 86+9.2 |  |
| Beaule PE | CORR | 2015 | PAO | 67 | 72 | 60 | 32.3 | - | 4 | - | - | 2 |
| Ricciardi BF | Hip international | 2016 | PAO | 77 | 82 | 23 | 25 | - | 0 | Mild Dysplasia:  58+12 | 86+12 | 6 |
|  |  |  |  |  |  |  |  |  |  | Severe Dysplasia:  61+14 | 84+16 |  |
| Matsunaga A | Int Orthopaedics | 2018 | PAO | 113 | 113 | 51.2 | 38.3 | 22.1 | 5 | Delayed Union: 77.0+8.2 | 91.2+8.3 | 19 |
|  |  |  |  |  |  |  |  |  |  | Union: 75.8+11 | 93.8+5.5 |  |
| Yang J | Int Orthopaedics | 2018 | PAO | 643 | - | 12 | 26 | - | 0 | - | - | 8 |
| Biedermann E | Int Orthopaedics | 2008 | PAO | 50 | 60 | 88.8 | 27.3 | - | 39 | - | - | 47 |
| Seo H | JBJS | 2018 | PAO | 92 | 95 | 57.6 | 38.9 | 22 | 5 | 78.0+9.0 | 17.0+10.0 | 14 |
| Wells J | JBJS | 2018 | PAO | 206 | 238 | 123.6 | 26 | 24 | 0 | 66.0+14.0 | 92.4+8.4 | 12 |
| Clohisy JC | JBJS | 2017 | PAO | 391 | 391 | 31.2 | 25.4 | 24.9 | 12 | 61.2 | 85.1 | 26 |
| Hartig-Andreasen C | JHPS | 2017 | PAO | - | 55 | 42 | 36.1 | - | 14 | 63.8+6.9 | 65.7+5.0 | - |
| Thanacharoenpanich S | JHPS | 2018 | PAO | - | 107 | 25 | 28 | 24.9 | 10 | 61.5+15.1 | 84.5+14.5 | - |
| Ricciardi BF | AJSM | 2016 | PAO | 77 | 87 | 15 | 23.7 | 22.6 | 2 | Post-Scope:58+13 | 83.0+14.0 | 6 |
|  |  |  |  |  |  |  |  |  |  | No Scope: 61+14 | 84.0+16 |  |
| Ricciardi BF | AJSM | 2017 | PAO | 93 | 110 | 24 | 23 | 22.5 | 0 | Post-Scope:54+16 | 76.0+15.0 | 10 |
|  |  |  |  |  |  |  |  |  |  | No Scope: 59+13 | 85.0+15 |  |
| Sabbag CM | AJSM | 2019 | PAO | 240 | 248 | 36 | 26.6 | - | 13 | - | - | 24 |
| Novais EN | JBJS | 2015 | PAO | 280 | - | 48 | 33.4 | - | 0 | - | - | 54 |
| Wasko MK | JBJS | 2019 | PAO | 294 | - | 24 | 21 | 23.4 | 0 | 60.0+14.8 | 82.9+16.5 | - |
| Yuasa T | Archives of Orthopaedic and Trauma Surgery | 2017 | RAO | 156 | 178 | 244 | 41.4 | - | 34 | Pre-coxathrosis:  62.9+13.2 | 83.3+8.88 | - |
|  |  |  |  |  |  |  |  |  |  | Initial coxarthrosis: 68.4+14.4 | 87.4+8.5 |  |
|  |  |  |  |  |  |  |  |  |  | Advanced Coxarthrosis: 65.8+14.4 | 71.2+12.7 |  |
| Tomioka M | BMC Musculoskeletal Disorders | 2017 | RAO | 56 | 65 | 180 | 36.5 | 21.2 | 0 | - | - | 0 |
| Okano K | CORR | 2008 | RAO | 47 | 49 | 147.6 | 43.1 | - | 0 | - | - | 0 |
| Okano K | CORR | 2010 | RAO | 89 | 92 | 146.4 | 32.5 | - | 0 | - | - | 6 |
| Maruyama M | CORR | 2013 | RAO | 62 | 71 | 60 | 39.7 | 22.9 | 6 | - | - | 17 |
| Yasunaga Y | CORR | 2016 | RAO | 159 | 173 | 252 | 35.1 | - | 0 | - | - | - |
| Min BW | Clinics in Orthopedic Surgery | 2018 | RAO | 64 | 71 | 207.6 | 39 | 22.8 | 8 | 71.8+7.5 | 85.1+9.0 | 2 |
| Irie T | Hip International | 2017 | RAO | 17 | 19 | 64.8 | 37.8 | - | 0 | 62.1+11.0 | 89.7+23.3 | - |
| Nozawa M | HSS Journal | 2009 | RAO | 351 | 420 | 131.7 | 26.8 | - | 2 | - | - | 0 |
| Nozawa M | Int Orthopaedics | 2008 | RAO | 161 | 179 | 148.6 | 45.2 | - | 0 | - | - | 0 |
| Kanezaki S | Int Orthopaedics | 2017 | RAO | 78 | 87 | 99.6 | 36 | - | 9 | - | - | 3 |
| Karasuyama K | Journal of Orthopaedic Surgery and Research | 2018 | RAO | 127 | 147 | 106.8 | 39.8 | 22.98 | 11 | - | - | 11 |
| Yasunaga Y | BJJ | 2019 | RAO | 31 | 37 | 214.8 | 17.4 | - | 0 | 73 | 91 | 1 |
| Kaneuji A | JBJS | 2015 | RAO | 91 | 93 | 270 | 32.4 | - | 14 | Pre-OA: 76.6+11.6 | 89.4+12.9 | - |
|  |  |  |  |  |  |  |  |  |  | Intermediate OA: 74.9+12.0 | 84.5+19.0 |  |
|  |  |  |  |  |  |  |  |  |  | Advanced OA: 65.1+13.2 | 84.1+11.0 |  |
