# Supplementary figures and images for: Pelvic osteotomies for acetabular dysplasia: Are there outcomes, survivorship and complication differences between different osteotomy techniques?
Source: J Hip Preserv Surg. 2021 Feb 5;7(4):764–76. doi: 10.1093/jhps/hnab009 (PMC8349594; doi:10.1093/jhps/hnab009)

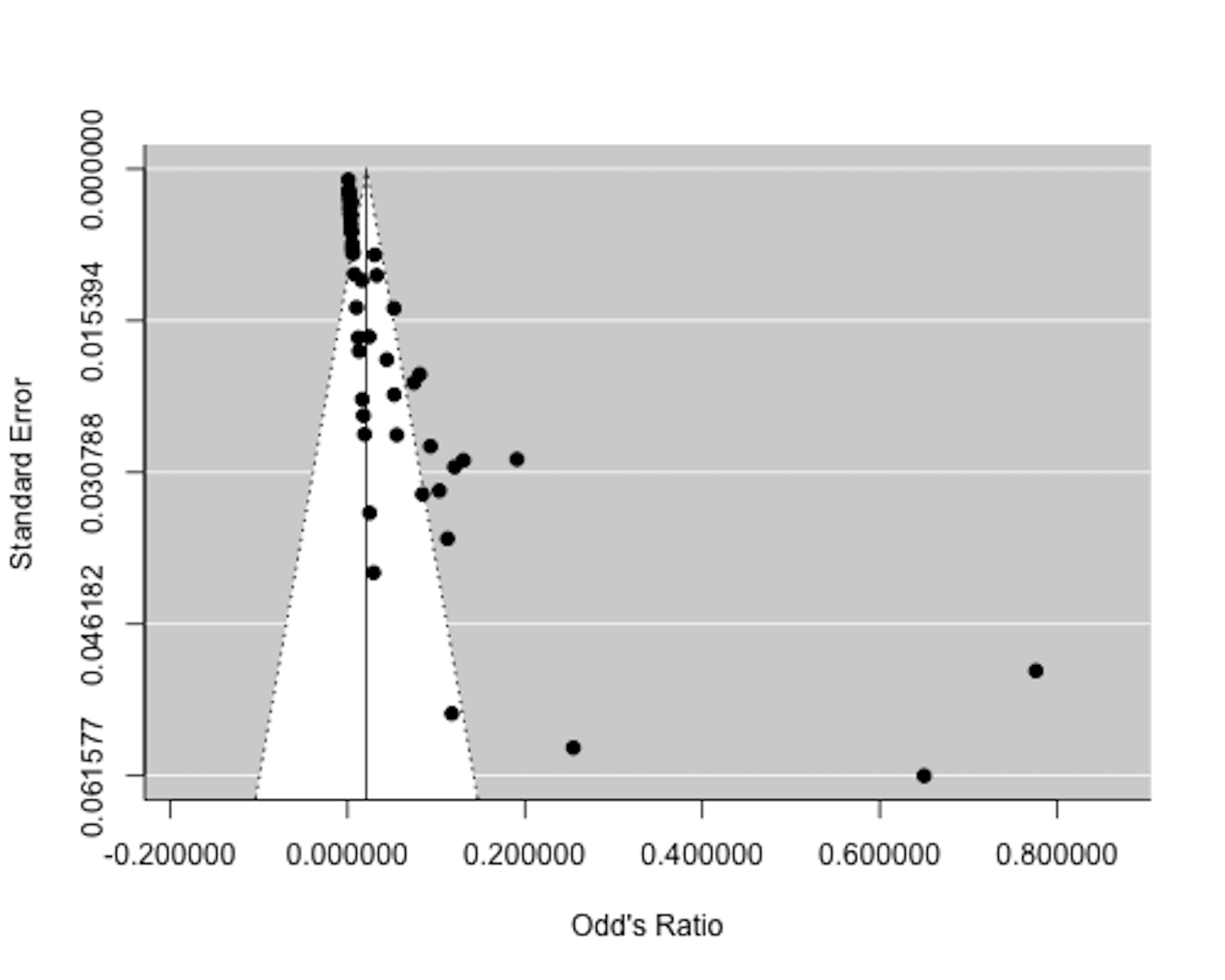

Supplement: hnab009_Supplementary_Data [file hnab009_supplementary_data.zip › hnab009-suppl_data/Appendix II.png]
